# Effects of Radiotherapy in the treatment of multiple myeloma: a retrospective analysis of a Single Institution

**DOI:** 10.1186/s13014-015-0374-z

**Published:** 2015-03-28

**Authors:** Christiane Matuschek, Thomas A Ochtrop, Edwin Bölke, Ute Ganswindt, Roland Fenk, Stephan Gripp, Patric Kröpil, Peter Arne Gerber, Kai Kammers, Jackson Hamilton, Klaus Orth, Wilfried Budach

**Affiliations:** Department of Radiation Oncology, Faculty of Medicine, Heinrich-Heine-University Düsseldorf, Moorenstraße 5, 40225 Düsseldorf, Germany; Department of Hematology Oncology and Clinical Immunology, Faculty of Medicine, Heinrich-Heine-University Düsseldorf, Dusseldorf, Germany; Department of Diagnostic and Interventional Radiology, Faculty of Medicine, Heinrich-Heine-University Düsseldorf, Dusseldorf, Germany; Department of Dermatology, Faculty of Medicine, Heinrich-Heine-University Düsseldorf, Dusseldorf, Germany; Department of Biostatistics, Johns Hopkins Bloomberg School of Public Health, Baltimore, MD USA; Faculty of Diagnostic Radiology, The University of Texas MD Anderson Cancer Center, Houston, Texas USA; Department of General, Medical Faculty, Visceral, and Thoracic Surgery, Asklepios Harz Hospitals, Goslar, Germany; Department of Radiation Oncology, Faculty of Medicine, University of Munich (LMU), Munich, Germany

**Keywords:** Multiple myeloma, Plasmocytoma, Radiotherapy, Radiation therapy, Analgesic effect, Recalcification, Side effects

## Abstract

**Background:**

Palliative irradiation of osteolytic lesions is a considerable component in the treatment for patients with multiple myeloma. In this study, we analyzed the efficacy of irradiation in these patients.

**Patients and methods:**

We retrospectively analyzed 153 patients with multiple myeloma who were admitted to our department between 1989 and 2013. According to the staging system of Durie & Salmon 116 patients were classified as stage III. 107/153 patients were treated with radiotherapy of at least one and up to 6 bony lesions at different times. In order to evaluate the effect of local radiotherapy on pain relief and bone recalcification a uni- and multivariate analysis was performed using a binary logistic regression model to correct for multiple measurements. Complete information on dose, fractionation and volume of radiotherapy was available from 81 patients treated in 136 target volumes for pain relief, and from 69 patients treated in 108 target volumes for recalcification. Total radiation doses varied between 8 Gy to 50 Gy (median dose 25 Gy in 2.5 Gy fractions, 5 times a week).

**Results:**

Radiotherapy resulted in complete local pain relief in 31% and partial local pain relief in 54% of the patients. In the univariate analysis, higher total radiation doses (p = 0.023) and higher age (p = 0.014) at the time of radiotherapy were significantly associated with a higher likelihood of pain relief, whereas no significant association was detected for concurrent systemic treatment, type and stage of myeloma and location of bone lesions. The same variables were independent predictors for pain relief in the multivariate analysis. Recalcification was observed in 48% of irradiated bone lesions. In the uni- and multivariate analysis higher radiation doses were significantly associated (p = 0.048) with an increased likelihood of recalcification. Side effects of radiotherapy were generally mild.

**Conclusions:**

Higher total biological radiation doses were associated with better pain relief and recalcification in this retrospective evaluation of multiple myeloma patients. In addition, in the elderly the therapeutic measures appear to develop a better analgesic effect.

## Introduction

Multiple myeloma is a cancer of plasma cells, clinically characterized by recurrent bone pain, soft tissue masses, anemia, infections, neurological symptoms, hypercalcemia and renal failure. Modern treatment of multiple myeloma consists of a combination of different treatment approaches including high-dose chemotherapy followed by autologous stem cell transplantation, immunomodulatory drugs, such as thalidomide, lenalidomide, pomalidomide and proteasome inhibitors, such as bortezomib, carfilzomib or MLN9708. With these novel combinations the median overall survival of patients with multiple myeloma approaches 50% and better, 10 years after first diagnosis [1]. Although these are rare tumors they are the major causes of bone involvement [2]. Osteolytic process leads to an increased risk of pathologic fracture and severe pain with negative impact on quality of life.

Palliative irradiation of painful osteolytic processes is an important component in the treatment of multiple myeloma [3]. The efficacy of different radiation regimens in the palliative treatment of bone metastases from solid tumors has been tested in a number of trials [4-7]. In some trials a small number of patients with multiple myeloma were included, but in most cases no subgroup analysis was presented [7]. As a consequence, different consultants used different radiation regimens ranging from 1×8 Gy to 20×2 Gy in the palliative treatment of painful bone lesions and in patients with multiple myeloma. In our retrospective study, we analyzed the effects of these different radiation regimes on pain relief and recalcification.

## Materials and methods

We retrospectively collected data from the records of patients with the diagnosis of multiple myeloma treated by the Departments of Radiation Oncology and Hemato-Oncology at the Heinrich-Heine-University in Düsseldorf, Germany, from 1989 to 2013. Patients were excluded, if their documentation lacked accurate information or they suffered from another acutely life threatening neoplasm. 153 patients were identified of whom 107 underwent radiotherapy and of whom 46 patients did not receive radiotherapy at any time. Patients’ characteristics are displayed in Tables 1 and 2. Complete information on dose, fractionation and volume of radiotherapy was available in 99 patients. Patients were followed until 2013. For the end point pain relief after radiotherapy, valid data from 81 patients treated in 136 targeted volumes were available (1–6 target volumes per patient, median: 1 volume). For the end point recalcification after radiotherapy, valid data of 69 patients treated in 108 targeted volumes were available (1–5 target volumes per patient, median: 1 volume). Fractionated radiotherapy was administered in 4–5 fractions per week. Most target volumes were irradiated with 6–25 MV photons; a minority of 29 target volumes was treated with a cobalt-60 device. External beam radiation was delivered via one to three or greater external beams. Indications for radiotherapy were osseous pain, pathologic fractures, or neurological symptoms related to osteolytic lesions. On average, radiotherapy was performed 4 months after initial diagnosis, ranging from 1 to 179 months. Systemic therapy was given simultaneously to 108 of the irradiated patients (Table 1). Surgical intervention in the area of irradiation was performed in 46 patients (19 at peripheral fractures and 27 vertebral operations, e.g. 9 vertebroplastics, 11 laminectomies and 7 other procedures). The follow-up was 1 up to 22 years after completing radiotherapy treatment in different time intervals.

**Table 1:** **Patients’ characteristics**

**Characteristics**		**N**
Irradiation	Irradiated Pat.	107 (70%)
	Target Vol.	202
	No Radiation	46 (30%)
Status	Alive	77 (50%)
Sex	Male	86 (56%)
	Female	67 (44%)
Age (years)	Range 30-87	59 median
Classification	IgG kappa	59 (39%)
	IgG lambda	25 (16%)
	IgA kappa	21 (14%)
	IgA lambda	15 (10%)
	Kappa	15 (10%)
	Lambda	05 (03%)
	Diverse	13 (08%)
Stage at first diagnosis	I	21 (14%)
(Durie&Salmon [8])	II	16 (10%)
	III A	99 (65 %)
	III B	17 (11%)
Radiation dose (Gy)	08	02 (01%)
	20	63 (31%)
	25	36 (18%)
	30	26 (13%)
	36	38 (19%)
	40	26 (13%)
	Diverse	11 (05%)
Simultaneous chemotherapy	(with statement about analgesia)	108 (73)
Surgical intervention		29 (20)

n = number of patients, Stage = Durie and Salmon.

**Table 2:** **Anatomic location of irradiated osteolyses**

**Location**			**Analgesia %**			**Side Effects %**
		**No.**	**Total**	**Full**	**Partial**	**(Level I-II)**
**Spine**	Thoracic Spine (TS)	32	84	38	47	20
	Lumbar Spine (LS)	12	100	25	75	25
	Cervical Spine (CS)	8	75	38	37	13
	CS/TS	4	100	100	0	0
	TS/LS	11	79	45	54	20
**Extremities**	Superior	12	91	16	75	16
	Inferior	15	80	0	80	8
**Trunk**	Pelvis/Hip	24	87	41	45	19
**Others**	Sternum	3	100	10	90	25
	Ribs	9	55	11	44	11
	Clivus	2	100	50	50	0
	Skull	10	70	10	60	50
**Extramedullary tumor**		10	90	30	60	21
**Other tocations/missing data**		18				

Level (RTOG).

Analgesic effects during the first year after radiotherapy were retrospectively extracted from the patient’s files using a Likert scale [9] for pain. The analgesic effect was categorized to complete pain relief, partial pain relief and no pain relief. For the statistical analysis a binary outcome was used taking together partial to complete pain relief to one category.

Recalcification was based on assessment of tumor remission by World Health Organization (WHO) criteria and divided into 4 categories of skeletal metastases [10]: complete remission (CR, for at least 4 weeks), partial remission (PR, size reduction and recalcification), no change (status idem), and progressive disease (PD), which was defined as a size increase of more than 20% [11]. Recalcification was measured with the help of a computed tomography (CT) [12]. Degree of recalcification was based on a comparison of pre-treatment and post-treatment (at least 3 months to 1 year after irradiation) radiographs (CT and/or MRI) using ROI (Region Of Interest) and diameter measurement, as well as manifest sclerosis. For the statistical analysis a binary outcome was used taking partial to complete remission to one category and no change and progression to the other category.

In addition, available information in radiation related side effects were recorded and categorized by the criteria of Radiation Therapy Oncology Group (RTOG) [13]. Overall survival was also assessed up to the year of 2013 for all patients.

### Statistical analysis

For statistical analysis, SPSS 21.0 was used. Frequencies were determined by descriptive analyses. P-values <0.05 were regarded as statistically significant. Overall survival was estimated by the Kaplan-Meier method and differences of subgroups were tested by the log-rank test.

In irradiated patients, the effects of radiotherapy on the end points pain relief and recalcification were assessed by using an uni- and multivariate binary logistic regression model that incorporates adjustments for repeated measurements in the same patient (GENLIN procedure, SPSS 21.0). Radiation dose and fractionation was normalized according to the linear quadratic model [14] assuming no relevant repopulation to 2 Gy equivalence doses for α/β-values of 2 Gy and 10 Gy. The Wald test was employed to assess the significance of tested variables and for exclusion from the model in the stepwise backward procedure in the multivariate analysis. Since 2 Gy equivalence doses using α/β-values of 2 Gy and 10 Gy were highly co-correlated, the initial model of the multivariate analysis included the 2 Gy equivalence dose using an α/β-values 10 Gy as dose variable only, which was the better predictor of dose response for both end points (pain relief and recalcification).

## Results

### Analgesia

**Figure 1:**
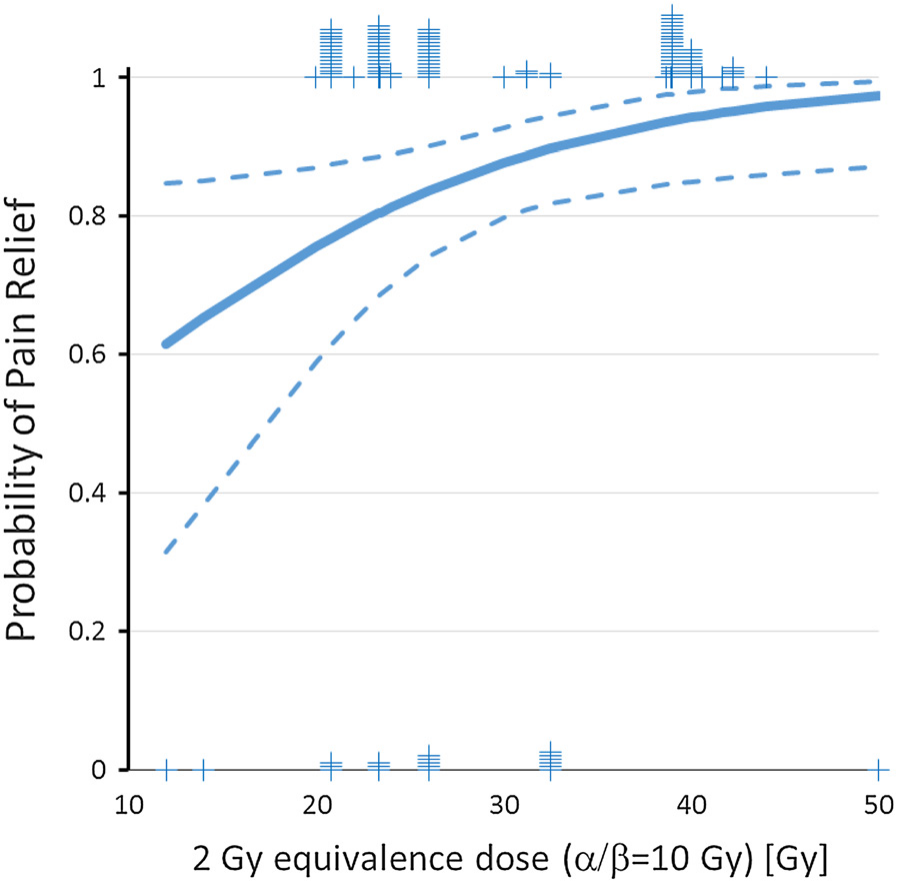
**Binary logistic regression analysis of dose effects on pain relief (α/β = 10 Gy, p =0.023.** Dotted lines indicate the 95% confidence limits of the regression line. Tick marks indicate the number of events (0 or 1) at the respective dose.

**Figure 2:**
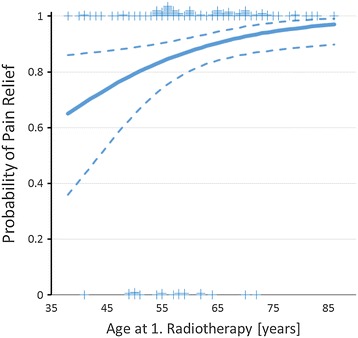
**Binary logistic regression analysis of age at 1st. radiotherapy on pain relief (α/β = 10 Gy, p = 0.014.** Dotted lines indicate the 95% confidence limits of the regression line. Tick marks indicate the number of events (0 or 1) at the respective dose.

Treatment-related analgesia occurred during or at the end of the course of radiotherapy. In follow-up records we noticed pain relief in 85% of the patients which lasted for at least 1 year after radiotherapy. We evaluated 136 target volumes in 81 patients in terms of analgesia. In 85% of the treated patients, an analgesic effect was achieved by irradiation of painful areas. Patients reported complete and partial pain relief in 31% and 54% of the treated lesions, respectively (no change = 12%, progression = 3%). In the uni- and multivariate binary logistic regression analysis improved pain relief (complete/partial) was significantly associated with higher total 2 Gy-equivalence doses (α/β = 10 Gy) and higher patient’s age at the time of radiotherapy, whereas all other tested factors were not associated with pain relief (Table 3). According to the model, an increase from 20 to 30 Gy total dose (2 Gy equivalence (α/β = 10 Gy) resulted in a 12% higher likelihood of complete or partial pain relief (Figure 1). Also, a 70 year old patient showed a 13% greater chance of complete or partial pain relief after radiotherapy compared to a 50 year old patient (Figure 2). Furthermore we found no correlation between pain relief and recalcification (Chi square test, p = 0.35).

**Table 3:** **Univariate and multivariate analysis of analgesic effect and recalcification of patients undergoing radiotherapy**

**End point**	**Variable**	**Exp (B)**	**Lower 95% CL of Exp (B)**	**Upper 95% CL Exp (B)**	**p-value**
Pain relief	α/β =2 Gy	1.072	1.002	1.146	0.043*
Pain relief	α/β =10 Gy	1.087	1.012	1.168	0.023*
Pain relief	target volume	1.088	1.015	1.167	0.018*
Pain relief	age at RT	1.062	1.012	1.114	0.014*
Pain relief	gender	1.432	0.469	4.376	0.529
Pain relief	Typ kappa	2.572	0.685	9.654	0.162
Pain relief	Typ lambda	0.360	0.077	1.676	0.193
Pain relief	IgG	1.228	0.405	3.702	0.717
Pain relief	IgA	1.91	0.253	3.312	0.893
Pain relief	concurrent systemic treatment (yes/no)	1.285	0.241	6.863	0.769
Recalcification	α/β =2 Gy	1.043	0.996	1.091	0.074
Recalcification	α/β =10 Gy	1.050	1.000	1.101	0.048*
Recalcification	target volume	1.049	1.000	1.100	0.050*
Recalcification	age at RT	1.023	0.983	1.064	0.272
**Multivariate analysis**					
Pain relief	α/β =10 Gy	1.087	1.007	1.172	0.032*
Pain relief	age at RT	1.066	1.008	1.127	0.025*
Recalcification	α/β =10 Gy	1.050	1.000	1.101	0.048*

CL Confidence Level, *significance.

Univariate Analysis.

### Recalcification

**Figure 3:**
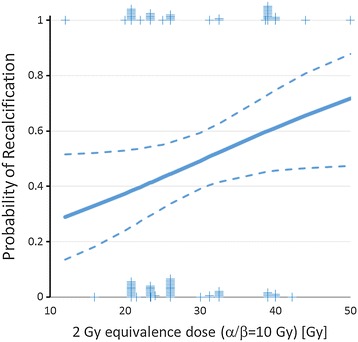
**Binary logistic regression analysis of dose effects on recalcification (**α**/**β **= 10 Gy, p = 0.048.** Dotted lines indicate the 95% confidence limits of the regression line. Tick marks indicate the number of events (0 or 1) at the respective dose.

The effect of radiotherapy in 108 target volumes of 69 patients were evaluated for change in calcification based on pre- and post-treatment radiographs. Overall, recalcification was observed in 48% of the treated bone lesions. In 23% of cases full recalcification and in 25% partial recalcification was observed. No change of calcification could be documented in 42% of the bone lesions, and 10% of the patients developed progression of osteolytic lesions. In the uni- and multivariate binary logistic regression analysis higher total (2 Gy-equivalence) doses (α/β = 10 Gy) were significantly associated with better recalcification (complete/partial), whereas all other tested factors were not associated with better recalcification (Tables 2,3,4). According to the model, an increase from 20 to 30 Gy total dose (2 Gy equivalence dose, α/β = 10 Gy) resulted in a 12% higher likelihood (p = 0.048) of complete or partial recalcification (Figure 3).

**Table 4:** **Analgesic effect and remineralization of patients undergoing radiotherapy**

			**All**	**Effective Treatment**	
			**n**	**n %**	
**Analgesia**	Simultaneous Chemotherapy	Yes	73	64	87.7%
		No	66	55	83.3%
	Surgical Intervention	Yes	20	16	80.0%
		No	119	103	86.6%
**Remineralization**	Simultaneous Chemotherapy	Yes	65	29	44.6%
		No	44	26	59.1%
	Surgical	Yes	19	07	36.8%
	Intervention	No	90	48	53.3%

n = number of patients.

### Side effects

In this retrospectively evaluated cohort, 40 of 107 patients (37%) suffered from side effects related to radiotherapy. Of these, 50% showed grade 1, and 47.2% grade 2 side effects. One patient suffered from dysphagia (grade 3 adverse event). No case of radiation induced myelopathy was observed clinically.

### Survival

**Figure 4:**
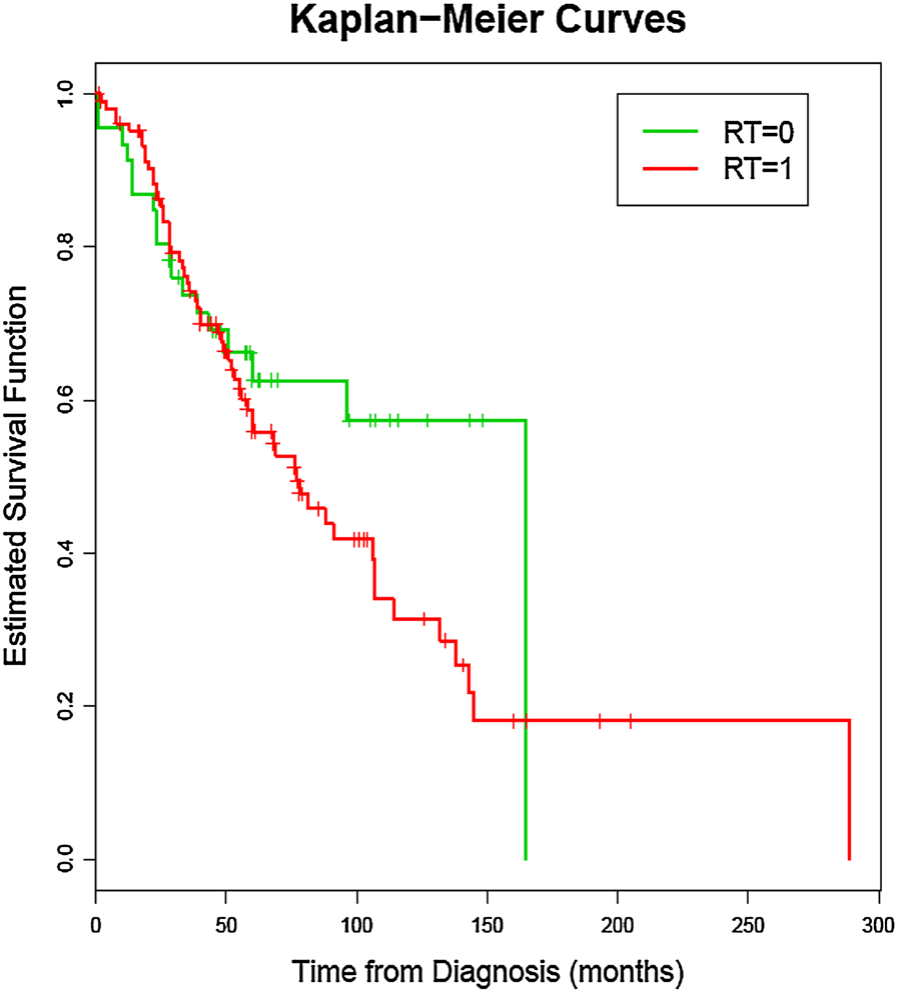
**Kaplan-Meier Curves; RT = 0, for patients not treated by radiotherapy and RT = 1, for patients treated with radiotherapy, p = 0.233.**

Median overall survival was 89.1 months for the whole cohort. Patients requiring palliative radiotherapy for painful bone involvement had a worse prognosis than patients without these findings (Figure 4) (median survival time 77 vs. 165 months, p = 0.233). But there was no significant difference.

## Discussion

### Pain relief

Osteolytic lesions and bone pain is a common symptom in patients with multiple myeloma and present at the time of first diagnosis in 70% of cases [5]. Larger osteolytic lesions are frequently associated with pain and the risk of fracture. Accordingly, radiotherapy in addition to systemic treatment is typically administered in these situations and is frequently associated with rapid pain relief. Bone disease remains complex and is caused by the production of the osteoclast stimulating enzymes [15]. The inhibition of pain mediators and the shrinkage of the tumor are thought to be the main mechanisms of analgesic effects derived from irradiation. The often rapid analgesic effect of irradiation is not completely understood. Since ionizing irradiation induces apoptosis in myeloma cells within 72 h [16], rapid death of myeloma cells resulting in decompression of nerves and pressure sensors following tumor shrinkage is probably the most important mechanism. Other proposed mechanisms include the obstruction of the secretion of mediators such as substance P and cytokines, at the interface of myeloma cells and the bone matrix [17,18]. Recalcification is achieved long term after a few months, while an analgesic effect is obtained during or immediately after radiotherapy.

The analgesic success rate of radiotherapy in our study was 85% of all irradiations. In the period of aftercare, 54% of our patients achieved partial analgesic effect and 31% achieved complete pain relief. Even partial pain relief is appropriate in view of the few side effects of radiotherapy. Uni- and multivariate logistic regression analysis revealed significant better pain relief at increased (2 Gy equivalence) total dose (α/β = 10 Gy, Figure 1) and with increasing age at time of radiotherapy (Figure 2). Using a α/β of 10 Gy to calculate 2 Gy equivalent total doses resulted in steeper dose response curves than using a α/β of 2 Gy for calculation indirectly indicating that pain relief behaves radiobiologically like an acute effect.

The high efficacy of radiotherapy that induces complete or partial pain relief in 75-95% of patients with painful myeloma bone lesions has been reported from several independent retrospective evaluations [7,19,20]. The majority of the patients in these investigations were treated with total doses between 30 and 40 Gy, typically using fraction sizes between 2 and 3 Gy. Relatively few patients were treated with low doses like 1×8 Gy or 5×4 Gy. With a few exceptions, investigators did not test whether greater radiation doses are associated with improved pain control, either because of too small variation in the employed radiation schedules or because of too short variation in pain control. Stölting et al. found in a univariate analysis significantly better pain control for total doses between 40–49.9 Gy compared to total doses below 30 Gy as well as for doses per fraction of 2 Gy compared to doses per fraction of ≥4 Gy. In the multivariate analysis, only the use of 2 Gy per fraction compared to doses per fraction of ≥4 Gy remained significant. Since 2 Gy per fraction schedules were highly associated with total doses of 40 Gy or greater, the reported results can be interpreted as evidence for a significant dose response relationship. Unfortunately, the authors did not normalize their different fractionation schedule by using 2 Gy equivalent dose or normalized total doses, making a direct comparison to the data presented here difficult. Leigh et al. [21] reported a slight trend to higher rates of pain relief at higher radiation doses, but did also not employ a normalization of the used fractionation schedule. In the work of Adamietz et al. [8] the median total radiation doses (not normalized) of responding and not responding myeloma patients in terms of pain relief were identical, indirectly indicating no distinct dose response relationship. Whether the use of normalized total doses and more advanced statistical tools would have led to the detection of significant dose response relationships for pain control, however, remains unclear. In view of the retrospective nature of the data presented here and the relatively small number of patients treated with normalized total doses below 20 Gy, the question, which dose and fractionation is most appropriate for painful bone lesions from multiple myeloma remains unresolved. Additionally, all patients included in our analysis received anti-osteoclastic drugs such as bisphosphonates as well as analgetics, if necessary The associated effects of these treatments are difficult to account for given their universal use. In our investigation we found no correlation between pain relief and recalcification. Adamietz et al. [8] reported a significantly longer duration of pain control at higher total radiation doses. No data in this regard are available from other authors.

An unexpected finding was that higher age at radiotherapy was associated with better pain control. This is in contradiction to the results of Stölting et al. [7], who reported better outcome in patients <60 years compared to patients >70 years. Using the same dichotomous categories instead of age as continuous variable, we found no age effect in our cohort (data not shown). The considerably younger average age in the cohort presents here may explain the different findings that otherwise remain unexplained. Probably the analgesic effect is a summation of the previous treatment, which depends on the biology of the tumor and the prior chemotherapy.

The effect of systemic treatments on pain relief in patients with ostelytic bone lesion has not been well investigated. The shape of the dose response curve for radiotherapy (Figure 1) suggests that approximately 50% of patients may have the chance to experience pain relief without radiotherapy most likely as result of the effect of systemic treatments. Concurrent systemic therapy at the time of radiotherapy was associated with better pain control in some reports [6]. This association was not confirmed in the present investigation. Since no details on concurrent and sequential systemic treatments were reported from any of the analyzed cohorts, potential interaction of radiotherapy with systemic treatments cannot be reliably performed. However, very few severe side effects have been reported, indicating that important interactions of radiotherapy and systemic treatments used for the treatment of multiple myelomas are probably rare.

### Recalcification

Beside pain relief, a recalcification of bony lesions is desirable to reduce the bone fracture rate. Therapy of solitary plasmocytoma underline higher target volume doses are more effective. Total doses of 45 Gy or higher in 2.0-2.5 Gy per fraction seem to eradicate most tumors [22,23] and is re-emphasized by studies of solid tumors [19]. But these clinical trials do not examine multiple myeloma osteolyses in particular.

The recalcification success rate of radiotherapy in our study was 48% of all irradiations. During follow up, 25% of our patients achieved partial recalcification and 23% reached complete recalcification. Uni- and multivariate logistic regression analysis revealed a significant better recalcification at increased (2 Gy equivalence) total dose (α/β = 10 Gy, Figure 3), whereas all other tested variables were not predictive for the likelihood of recalcification (Table 4). A limitation of our analysis is that it includes sole radiological reports and/or ROI and diameter measurement of the levels of recalcification, e.g. sclerosis, with the help of radiographs (CT, MRI or conventional X-ray imaging).

Recalcification after radiotherapy has been reported in 11-50% of patients with bone lesions of solid tumors from several independent retrospective evaluations [6,7]. The majority of the patients in these investigations were treated with total doses between 30 and 50 Gy typically using fraction sizes between 2 and 3 Gy. Relatively few patients were treated with low total doses like 1×8 Gy or 5×4 Gy, and most studies analyzed osteolyses of diverse solid tumors, not just multiple myeloma patients. There were only few significant results found whether higher radiation doses are associated with improved recalcification for patients with multiple myeloma.

Koswig et al. [19] examined recalcification following radiation therapy with 2 different fractionation schedules (1 × 8 Gy vs 10 × 3 Gy) for bone metastasis of solid tumors. The recalcification showed a significant effect concerning patients in the fractionated group p <0.0001). In myeloma patients, Stölting et al. [7] found in a univariate analysis significant better recalcification for total doses of 50–60 Gy compared to total doses below 30 Gy. This association remained significant in the multivariate analysis. Further significant parameters for recalcification in the multivariate analysis were concurrent chemotherapy vs no chemotherapy, and no fractures vs fractures. The reported results can be interpreted as evidence for a significant dose response relationship. Unfortunately, the authors did not normalize total doses, making a direct comparison to the data presented here difficult. Balducci et al. [5] described recalcification in patients with osteolytic lesions due to diverse plasma cell neoplasm in 50% and identified as complete remission in 38%. Mose et al. [6] also found a relevant effect in concurrent chemotherapy, but no difference in recalcification in terms of radiation dose probably due to the low variability in total doses (30–36 Gy) (Table 5). In summary, available data indicate that higher radiation doses result in improved recalcification.

**Table 5:** **Remineralization of osteolysis at different radiation doses in literature**

	**n**	**Dose (range)Gy**	**Radiation schedule**	**Remineralization%**	**Status idem%**	**Progression of disease%**	**Period of measurement**
**Rieden 1986** **[** 26 **]**	7	40-50	5X2Gy	29	71	-	-
**Weber 1992** **[** 27 **]**	14	30-40	5X2Gy	71	29	-	-
**Liebross 1998** **[** 28 **]**	44	30-70	5X2Gy	43	-	-	-
**Norin 1957** **[** 29 **]**	53	12-48	-	51	-	-	1943-1953
**Mose 2000** **[** 6 **]**	56	18-45	5X2Gy	46	36	18	1988-1998
**Stoelting 2010** **[** 7 **]**	114	2-60	5X2Gy	45	49	16	1970-2003
***Current Data 2014***	108	20-60	diverse	48	42	10	1989-2014

n = number of patients, Gy = Gray, dose target volume.

### Side effects

Radiotherapy offers the advantage of few side effects and therefore is an appropriate palliative procedure for treating multiple myeloma [24,25]. Our analysis found 37% side effects with 50% grade 1, and 47.2% grade 2. One patient suffered from dysphagia (grade 3). A substantial increase in the side effects with simultaneous chemotherapy has not been reported. We found no correlation between radiotherapy-associated side effects and overall survival. Corresponding to us Foro and Arnalot [20] reported acute side effects in 18% and Balducci et al. [5] identified 44% of patients (n = 23) with side effects (grade 1–2): hematological toxicity in 11 (48%), gastroenteric toxicity in 6 (26%), pharyngeal toxicity in 2 (9%), and cutaneous toxicity in 4 (17%) patients. Mose et al. reported about 54% side effect mostly grade 1–2; in 4 % Grade 3 (hematopoietic changes, mucositis, creatinine level).

Median overall survival was 89.1 months for the whole cohort and radiation therapy statistically did not have an impact on survival.

## Conclusion

Palliative radiotherapy in plasma cell neoplasm’s mostly results in pain relief without significant toxicity. Our data indicate that higher total doses (30–36 Gy) are associated with improved pain relief. The analgesic effect of radiotherapy in myeloma patients appears to be less pronounced younger patients, indirectly indicating that higher radiation doses are especially beneficial in these patients. The likelihood of recalcification after radiotherapy is also increased at higher total radiation doses suggesting that higher doses (>40 Gy) should be considered, if recalcification is thought to be mandatory to lower the risk of fracture.
